# 2-Chloro-*N*-(4-chloro­benzo­yl)benzene­sulfonamide toluene hemisolvate

**DOI:** 10.1107/S1600536810052281

**Published:** 2010-12-18

**Authors:** P. A. Suchetan, Sabine Foro, B. Thimme Gowda

**Affiliations:** a Department of Chemistry, Mangalore University, Mangalagangotri 574 199, Mangalore, India; b Institute of Materials Science, Darmstadt University of Technology, Petersenstrasse 23, D-64287 Darmstadt, Germany

## Abstract

The unit cell of the title compound, C_13_H_9_Cl_2_NO_3_S·0.5C_7_H_8_, contains two mol­ecules of 2-chloro-*N*-(4-chloro­benzo­yl)benzene­sulfonamide and one toluene mol­ecule, which is disordered about a centre of inversion. The dihedral angle between the two aromatic rings is 85.7 (1)°. In the crystal, mol­ecules are linked by pairs of N—H⋯O(S) hydrogen bonds, forming centrosymmetric dimers.

## Related literature

For background to our study of the effect of ring and side-chain substitutions on the crystal structures of *N*-aryl sulfon­amides and for similar structures, see: Gowda *et al.* (2010*a* ▶,*b* ▶); Suchetan *et al.* (2010 ▶).
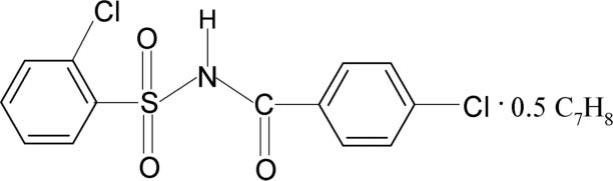


## Experimental

### 

#### Crystal data

C_13_H_9_Cl_2_NO_3_S·0.5C_7_H_8_
*M* *_r_* = 376.24
Triclinic, 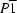

*a* = 7.5992 (9) Å
*b* = 10.876 (1) Å
*c* = 11.346 (1) Å
α = 73.868 (8)°
β = 75.927 (9)°
γ = 70.994 (8)°
*V* = 839.58 (15) Å^3^
*Z* = 2
Mo *K*α radiation
μ = 0.53 mm^−1^
*T* = 299 K
0.44 × 0.44 × 0.36 mm

#### Data collection

Oxford Diffraction Xcalibur diffractometer with a Sapphire CCD detector
Absorption correction: multi-scan (*CrysAlis RED*; Oxford Diffraction, 2009 ▶) *T*_min_ = 0.802, *T*_max_ = 0.834
5654 measured reflections
3405 independent reflections
2941 reflections with *I* > 2σ(*I*)
*R*_int_ = 0.009

#### Refinement

*R*[*F* ^2^ > 2σ(*F* ^2^)] = 0.039
*wR*(*F* ^2^) = 0.099
*S* = 1.06
3405 reflections
229 parameters
3 restraints
H atoms treated by a mixture of independent and constrained refinement
Δρ_max_ = 0.24 e Å^−3^
Δρ_min_ = −0.43 e Å^−3^

### 

Data collection: *CrysAlis CCD* (Oxford Diffraction, 2009 ▶); cell refinement: *CrysAlis RED* (Oxford Diffraction, 2009 ▶); data reduction: *CrysAlis RED*; program(s) used to solve structure: *SHELXS97* (Sheldrick, 2008 ▶); program(s) used to refine structure: *SHELXL97* (Sheldrick, 2008 ▶); molecular graphics: *PLATON* (Spek, 2009 ▶); software used to prepare material for publication: *SHELXL97*.

## Supplementary Material

Crystal structure: contains datablocks I, global. DOI: 10.1107/S1600536810052281/bt5432sup1.cif

Structure factors: contains datablocks I. DOI: 10.1107/S1600536810052281/bt5432Isup2.hkl

Additional supplementary materials:  crystallographic information; 3D view; checkCIF report

## Figures and Tables

**Table 1:** Hydrogen-bond geometry (Å, °)

*D*—H⋯*A*	*D*—H	H⋯*A*	*D*⋯*A*	*D*—H⋯*A*
N1—H1*N*⋯O1^i^	0.82 (2)	2.13 (2)	2.951 (2)	176 (2)

Symmetry code: (i) 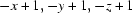.

**Fig. 1.**
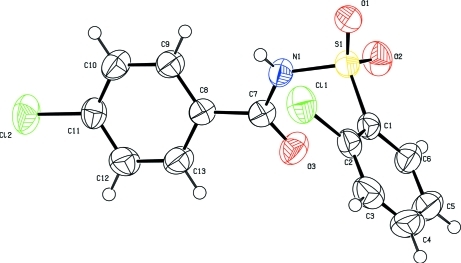
Molecular structure of the title compound, showing the atom-labelling scheme. Displacement ellipsoids are drawn at the 50% probability level.

**Fig. 2.**
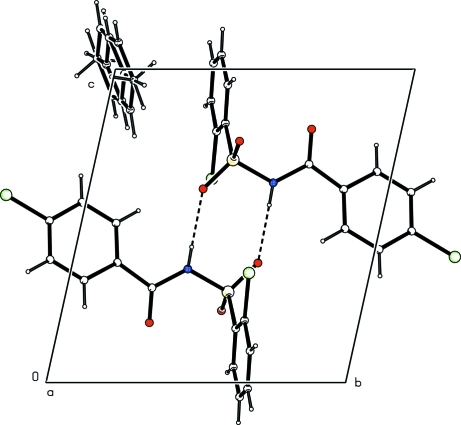
Molecular packing in the title compound. Hydrogen bonds are shown as dashed lines.

## supplementary crystallographic information

### Comment

In the present work, as a part of studying the effect of ring and the side chain substitutions on the crystal structures of *N*-aryl sulfonamides (Gowda *et al.*, 2010**a*,b*; Suchetan *et al.*, 2010), the structure of 2-chloro-*N*-(4-chlorobenzoyl)benzenesulfonamide (I) has been determined. The asymmetric unit of (I) contains also half a  molecule of toluene which is disordered about a centre of inversion. The conformation of the N—C bond in the C—SO_2_—NH—C(O) segment has *gauche* torsions with respect to the SO bonds. In these segments, the N—H bond is *anti* to the C=O bond (Fig. 1), similar to those observed in 2-chloro-*N*-(2-chlorobenzoyl)-benzenesulfonamide (II) (Suchetan *et al.*, 2010), 2-chloro-*N*-(3-chlorobenzoyl)benzenesulfonamide (III) (Gowda *et al.*, 2010*b*)), 2-methyl-*N*- (4-methylbenzoyl)benzenesulfonamide (IV) (Gowda *et al.*, 2010*a*).

The molecule in (I) is twisted at the *S* atom with the C—SO_2_—NH—C(O) torsional angle of -62.7 (2)°, compared to those of -66.5 (2)° in (II), -62.6 (3)° and -62.6 (2)° in the two molecules of (III), and -53.1 (2)° and 61.2 (2)° in the two independent molecules of (IV).

The dihedral angle between the sulfonyl benzene ring and the —SO_2_—NH—C—O segment is 88.5 (1)°, compared to the values of 86.9 (1) in (II), 89.9 (1)° and 86.4 (1)° in the two molecules of (III), and 86.0 (1)° (molecule 1) and 87.9 (1)° (molecule 2) in (IV).

Furthermore, the dihedral angle between the sulfonyl and the benzoyl benzene rings is 85.7 (1)°, compared to the values of 76.9 (1) in (II), 77.8 (1)° (molecule 1) and 83.5 (1)° (molecule 2) in (III), and 88.1 (1)° and 83.5 (1)° in the two independent molecules of (IV).

The packing of molecules linked by N—H···O hydrogen bonds (Table 1) is shown in Fig. 2.

### Experimental

The title compound was prepared by refluxing a mixture of 4-chlorobenzoic acid, 2-chlorobenzenesulfonamide and phosphorous oxychloride for 3 h on a water bath. The resultant mixture was cooled and poured into ice cold water. The solid obtained was filtered, washed thoroughly with water and then dissolved in sodium bicarbonate solution. The compound was later reprecipitated by acidifying the filtered solution with dilute HCl. It was filtered, dried and recrystallized.

Prism like colourless single crystals of the title compound used in X-ray diffraction studies were obtained by a slow evaporation of its toluene solution at room temperature.

### Refinement

The H atom of the NH group was located in a difference map and later restrained to N—H = 0.86 (1) %A. The other H atoms were positioned with idealized geometry using a riding model with C—H = 0.93 Å. All H atoms were refined with isotropic displacement parameters (set to 1.2 times of the *U*_eq_ of the parent atom). The solvent toluene molecule is disordered about a centre of inversion. The components of the displacement parameters of C15 C16 C17 were restrained to be equal within an effective standard deviation 0.01.

### Crystal data

**Table:**

C_13_H_9_Cl_2_NO_3_S·0.5C_7_H_8_	*Z* = 2
*M**_r_* = 376.24	*F*(000) = 386
Triclinic, *P*1	*D*_x_ = 1.488 Mg m^−^^3^
Hall symbol: -p 1	Mo *K*α radiation, λ = 0.71073 Å
*a* = 7.5992 (9) Å	Cell parameters from 3881 reflections
*b* = 10.876 (1) Å	θ = 2.9–27.8°
*c* = 11.346 (1) Å	µ = 0.53 mm^−^^1^
α = 73.868 (8)°	*T* = 299 K
β = 75.927 (9)°	Prism, colourless
γ = 70.994 (8)°	0.44 × 0.44 × 0.36 mm
*V* = 839.58 (15) Å^3^	

### Data collection

**Table:**

Oxford Diffraction Xcalibur diffractometer with a Sapphire CCD detector	3405 independent reflections
Radiation source: fine-focus sealed tube	2941 reflections with *I* > 2σ(*I*)
graphite	*R*_int_ = 0.009
Rotation method data acquisition using ω and φ scans	θ_max_ = 26.4°, θ_min_ = 2.9°
Absorption correction: multi-scan (*CrysAlis RED*; Oxford Diffraction, 2009)	*h* = −9→9
*T*_min_ = 0.802, *T*_max_ = 0.834	*k* = −13→13
5654 measured reflections	*l* = −13→14

### Refinement

**Table:**

Refinement on *F*^2^	Primary atom site location: structure-invariant direct methods
Least-squares matrix: full	Secondary atom site location: difference Fourier map
*R*[*F*^2^ > 2σ(*F*^2^)] = 0.039	Hydrogen site location: inferred from neighbouring sites
*wR*(*F*^2^) = 0.099	H atoms treated by a mixture of independent and constrained refinement
*S* = 1.06	*w* = 1/[σ^2^(*F*_o_^2^) + (0.0428*P*)^2^ + 0.4443*P*] where *P* = (*F*_o_^2^ + 2*F*_c_^2^)/3
3405 reflections	(Δ/σ)_max_ = 0.001
229 parameters	Δρ_max_ = 0.24 e Å^−^^3^
3 restraints	Δρ_min_ = −0.43 e Å^−^^3^

### Special details

**Table:**

Experimental. CrysAlis RED (Oxford Diffraction, 2009) Empirical absorption correction using spherical harmonics, implemented in SCALE3 ABSPACK scaling algorithm.
Geometry. All e.s.d.'s (except the e.s.d. in the dihedral angle between two l.s. planes) are estimated using the full covariance matrix. The cell e.s.d.'s are taken into account individually in the estimation of e.s.d.'s in distances, angles and torsion angles; correlations between e.s.d.'s in cell parameters are only used when they are defined by crystal symmetry. An approximate (isotropic) treatment of cell e.s.d.'s is used for estimating e.s.d.'s involving l.s. planes.
Refinement. Refinement of *F*^2^ against ALL reflections. The weighted *R*-factor *wR* and goodness of fit *S* are based on *F*^2^, conventional *R*-factors *R* are based on *F*, with *F* set to zero for negative *F*^2^. The threshold expression of *F*^2^ > σ(*F*^2^) is used only for calculating *R*-factors(gt) *etc*. and is not relevant to the choice of reflections for refinement. *R*-factors based on *F*^2^ are statistically about twice as large as those based on *F*, and *R*- factors based on ALL data will be even larger.

### Fractional atomic coordinates and isotropic or equivalent isotropic displacement parameters (Å^2^)

**Table:**

	*x*	*y*	*z*	*U*_iso_*/*U*_eq_	Occ. (<1)
Cl1	0.82406 (9)	0.59662 (6)	0.34975 (6)	0.06376 (18)	
Cl2	0.89482 (11)	−0.27177 (6)	0.59894 (6)	0.0726 (2)	
S1	0.46063 (7)	0.54050 (5)	0.28910 (4)	0.04093 (14)	
O1	0.4019 (2)	0.61891 (14)	0.38181 (13)	0.0523 (4)	
O2	0.3219 (2)	0.53244 (17)	0.22960 (15)	0.0594 (4)	
O3	0.6558 (3)	0.30065 (15)	0.19122 (13)	0.0597 (4)	
N1	0.5639 (2)	0.38940 (16)	0.36235 (15)	0.0428 (4)	
H1N	0.579 (3)	0.386 (2)	0.433 (2)	0.051*	
C1	0.6378 (3)	0.59331 (18)	0.17244 (17)	0.0395 (4)	
C2	0.7921 (3)	0.61655 (19)	0.19841 (19)	0.0456 (4)	
C3	0.9255 (3)	0.6578 (2)	0.1010 (3)	0.0635 (6)	
H3	1.0305	0.6722	0.1173	0.076*	
C4	0.9015 (4)	0.6773 (3)	−0.0197 (3)	0.0745 (8)	
H4	0.9911	0.7050	−0.0848	0.089*	
C5	0.7480 (4)	0.6567 (3)	−0.0455 (2)	0.0682 (7)	
H5	0.7325	0.6719	−0.1277	0.082*	
C6	0.6166 (3)	0.6134 (2)	0.04986 (19)	0.0523 (5)	
H6	0.5136	0.5977	0.0323	0.063*	
C7	0.6431 (3)	0.28362 (19)	0.30279 (18)	0.0421 (4)	
C8	0.7089 (3)	0.14941 (18)	0.38331 (18)	0.0404 (4)	
C9	0.6374 (3)	0.1159 (2)	0.50887 (19)	0.0485 (5)	
H9	0.5501	0.1807	0.5485	0.058*	
C10	0.6955 (3)	−0.0136 (2)	0.5754 (2)	0.0536 (5)	
H10	0.6468	−0.0362	0.6595	0.064*	
C11	0.8254 (3)	−0.1085 (2)	0.5167 (2)	0.0478 (5)	
C12	0.9002 (3)	−0.0773 (2)	0.3927 (2)	0.0581 (6)	
H12	0.9893	−0.1421	0.3541	0.070*	
C13	0.8411 (3)	0.0512 (2)	0.3268 (2)	0.0549 (5)	
H13	0.8906	0.0729	0.2427	0.066*	
C14	0.4143 (9)	−0.0335 (6)	1.0155 (6)	0.0821 (17)	0.50
C15	0.4774 (7)	0.0445 (4)	0.8986 (3)	0.0963 (11)	
H15A	0.4031	0.0715	0.8369	0.116*	0.50
H15B	0.5192	0.0831	0.8135	0.116*	0.50
C16	0.6245 (14)	0.0797 (6)	0.8707 (7)	0.1058 (17)	0.50
H16A	0.6644	0.1127	0.7870	0.127*	0.50
C17	0.7412 (8)	0.0691 (6)	0.9649 (7)	0.1470 (19)	
H17A	0.8390	0.1082	0.9492	0.176*	0.50
H17B	0.7570	0.1193	0.8808	0.176*	0.50
H17C	0.8486	−0.0073	0.9758	0.176*	0.50
H17D	0.7303	0.1239	1.0210	0.176*	0.50
C18	0.6696 (18)	−0.0094 (9)	1.0854 (11)	0.126 (4)	0.50
H18A	0.7398	−0.0358	1.1493	0.151*	0.50

### Atomic displacement parameters (Å^2^)

**Table:**

	*U*^11^	*U*^22^	*U*^33^	*U*^12^	*U*^13^	*U*^23^
Cl1	0.0641 (4)	0.0659 (4)	0.0699 (4)	−0.0150 (3)	−0.0330 (3)	−0.0130 (3)
Cl2	0.1049 (5)	0.0385 (3)	0.0687 (4)	−0.0145 (3)	−0.0255 (3)	0.0005 (3)
S1	0.0408 (2)	0.0379 (2)	0.0415 (3)	−0.00830 (18)	−0.00962 (19)	−0.00531 (19)
O1	0.0585 (9)	0.0427 (8)	0.0456 (8)	−0.0038 (6)	−0.0031 (6)	−0.0105 (6)
O2	0.0473 (8)	0.0649 (10)	0.0689 (10)	−0.0177 (7)	−0.0222 (7)	−0.0057 (8)
O3	0.0875 (12)	0.0494 (9)	0.0409 (8)	−0.0161 (8)	−0.0134 (7)	−0.0091 (6)
N1	0.0553 (10)	0.0353 (8)	0.0373 (8)	−0.0109 (7)	−0.0111 (7)	−0.0061 (7)
C1	0.0434 (10)	0.0313 (9)	0.0408 (9)	−0.0071 (7)	−0.0082 (8)	−0.0061 (7)
C2	0.0445 (10)	0.0347 (9)	0.0534 (11)	−0.0061 (8)	−0.0116 (9)	−0.0057 (8)
C3	0.0464 (12)	0.0493 (13)	0.0864 (18)	−0.0130 (10)	−0.0034 (11)	−0.0085 (12)
C4	0.0755 (17)	0.0581 (15)	0.0659 (16)	−0.0184 (13)	0.0197 (13)	−0.0034 (12)
C5	0.0890 (19)	0.0590 (15)	0.0431 (12)	−0.0150 (13)	0.0013 (12)	−0.0073 (10)
C6	0.0681 (14)	0.0454 (11)	0.0419 (11)	−0.0128 (10)	−0.0128 (10)	−0.0076 (9)
C7	0.0469 (10)	0.0404 (10)	0.0419 (10)	−0.0162 (8)	−0.0067 (8)	−0.0093 (8)
C8	0.0457 (10)	0.0373 (9)	0.0419 (10)	−0.0161 (8)	−0.0070 (8)	−0.0092 (8)
C9	0.0587 (12)	0.0393 (10)	0.0455 (11)	−0.0127 (9)	−0.0030 (9)	−0.0119 (8)
C10	0.0746 (15)	0.0443 (11)	0.0410 (11)	−0.0202 (10)	−0.0058 (10)	−0.0071 (9)
C11	0.0595 (12)	0.0348 (10)	0.0518 (11)	−0.0156 (9)	−0.0158 (9)	−0.0049 (8)
C12	0.0641 (14)	0.0410 (11)	0.0603 (13)	−0.0096 (10)	0.0025 (11)	−0.0143 (10)
C13	0.0694 (14)	0.0432 (11)	0.0449 (11)	−0.0159 (10)	0.0041 (10)	−0.0097 (9)
C14	0.078 (4)	0.068 (4)	0.085 (4)	0.015 (3)	−0.007 (3)	−0.041 (3)
C15	0.125 (3)	0.082 (2)	0.0583 (18)	0.014 (2)	−0.021 (2)	−0.0238 (17)
C16	0.146 (5)	0.051 (3)	0.079 (4)	−0.001 (4)	0.022 (3)	−0.016 (3)
C17	0.113 (4)	0.114 (4)	0.198 (6)	0.002 (3)	−0.018 (3)	−0.057 (4)
C18	0.164 (9)	0.088 (5)	0.154 (9)	−0.001 (6)	−0.108 (8)	−0.042 (5)

### Geometric parameters (Å, °)

**Table:**

Cl1—C2	1.737 (2)	C12—H12	0.9300
Cl2—C11	1.735 (2)	C13—H13	0.9300
S1—O2	1.4191 (15)	C14—C16^i^	1.248 (10)
S1—O1	1.4336 (15)	C14—C17^i^	1.311 (8)
S1—N1	1.6508 (17)	C14—C18^i^	1.342 (10)
S1—C1	1.763 (2)	C14—C15^i^	1.379 (8)
O3—C7	1.212 (2)	C14—C15	1.428 (8)
N1—C7	1.388 (2)	C14—C14^i^	1.613 (16)
N1—H1N	0.82 (2)	C15—C16	1.239 (10)
C1—C6	1.387 (3)	C15—C18^i^	1.252 (11)
C1—C2	1.389 (3)	C15—C14^i^	1.379 (8)
C2—C3	1.388 (3)	C15—H15A	0.9300
C3—C4	1.374 (4)	C15—H15B	0.9601
C3—H3	0.9300	C16—C14^i^	1.248 (10)
C4—C5	1.368 (4)	C16—C17	1.506 (11)
C4—H4	0.9300	C16—H15B	1.1326
C5—C6	1.373 (3)	C16—H16A	0.9300
C5—H5	0.9300	C16—H17B	1.2556
C6—H6	0.9300	C17—C14^i^	1.311 (8)
C7—C8	1.490 (3)	C17—C18	1.478 (13)
C8—C9	1.386 (3)	C17—H17A	0.9299
C8—C13	1.389 (3)	C17—H17B	0.9600
C9—C10	1.384 (3)	C17—H17C	0.9600
C9—H9	0.9300	C17—H17D	0.9599
C10—C11	1.371 (3)	C18—C15^i^	1.252 (11)
C10—H10	0.9300	C18—C14^i^	1.342 (10)
C11—C12	1.375 (3)	C18—H18A	0.9300
C12—C13	1.374 (3)		
			
O2—S1—O1	118.68 (10)	C18^i^—C14—C14^i^	106.8 (10)
O2—S1—N1	108.83 (9)	C15^i^—C14—C14^i^	56.4 (5)
O1—S1—N1	104.52 (9)	C15—C14—C14^i^	53.5 (5)
O2—S1—C1	107.90 (9)	C16—C15—C18^i^	173.8 (7)
O1—S1—C1	110.14 (9)	C16—C15—C14^i^	56.6 (5)
N1—S1—C1	106.05 (9)	C18^i^—C15—C14^i^	129.3 (7)
C7—N1—S1	122.20 (14)	C16—C15—C14	125.8 (6)
C7—N1—H1N	123.3 (17)	C18^i^—C15—C14	59.7 (6)
S1—N1—H1N	113.9 (17)	C14^i^—C15—C14	70.1 (6)
C6—C1—C2	119.90 (19)	C16—C15—H15A	116.1
C6—C1—S1	116.98 (16)	C18^i^—C15—H15A	58.4
C2—C1—S1	123.11 (15)	C14^i^—C15—H15A	167.6
C3—C2—C1	119.4 (2)	C14—C15—H15A	118.0
C3—C2—Cl1	118.42 (18)	C16—C15—H15B	60.4
C1—C2—Cl1	122.13 (16)	C18^i^—C15—H15B	113.7
C4—C3—C2	119.7 (2)	C14^i^—C15—H15B	117.0
C4—C3—H3	120.2	C14—C15—H15B	168.8
C2—C3—H3	120.2	H15A—C15—H15B	56.6
C5—C4—C3	121.0 (2)	C15—C16—C14^i^	67.3 (6)
C5—C4—H4	119.5	C15—C16—C17	123.2 (6)
C3—C4—H4	119.5	C14^i^—C16—C17	55.9 (5)
C4—C5—C6	120.0 (2)	C15—C16—H15B	47.5
C4—C5—H5	120.0	C14^i^—C16—H15B	114.8
C6—C5—H5	120.0	C17—C16—H15B	170.6
C5—C6—C1	120.0 (2)	C15—C16—H16A	117.5
C5—C6—H6	120.0	C14^i^—C16—H16A	174.9
C1—C6—H6	120.0	C17—C16—H16A	119.3
O3—C7—N1	121.13 (18)	H15B—C16—H16A	70.1
O3—C7—C8	122.36 (18)	C15—C16—H17B	160.2
N1—C7—C8	116.50 (16)	C14^i^—C16—H17B	94.6
C9—C8—C13	118.74 (18)	C17—C16—H17B	39.4
C9—C8—C7	123.58 (18)	H15B—C16—H17B	149.2
C13—C8—C7	117.56 (17)	H16A—C16—H17B	80.9
C10—C9—C8	120.19 (19)	C14^i^—C17—C18	57.1 (5)
C10—C9—H9	119.9	C14^i^—C17—C16	52.0 (5)
C8—C9—H9	119.9	C18—C17—C16	108.5 (6)
C11—C10—C9	119.68 (19)	C14^i^—C17—H17A	170.8
C11—C10—H10	120.2	C18—C17—H17A	126.6
C9—C10—H10	120.2	C16—C17—H17A	124.9
C10—C11—C12	121.18 (19)	C14^i^—C17—H17B	107.2
C10—C11—Cl2	119.84 (17)	C18—C17—H17B	164.3
C12—C11—Cl2	118.97 (17)	C16—C17—H17B	56.1
C11—C12—C13	119.0 (2)	H17A—C17—H17B	68.8
C11—C12—H12	120.5	C14^i^—C17—H17C	111.2
C13—C12—H12	120.5	C18—C17—H17C	79.3
C12—C13—C8	121.2 (2)	C16—C17—H17C	117.5
C12—C13—H13	119.4	H17A—C17—H17C	78.0
C8—C13—H13	119.4	H17B—C17—H17C	109.5
C16^i^—C14—C17^i^	72.1 (7)	C14^i^—C17—H17D	110.0
C16^i^—C14—C18^i^	138.6 (10)	C18—C17—H17D	78.3
C17^i^—C14—C18^i^	67.7 (7)	C16—C17—H17D	133.0
C16^i^—C14—C15^i^	56.1 (6)	H17A—C17—H17D	65.1
C17^i^—C14—C15^i^	128.1 (7)	H17B—C17—H17D	109.5
C18^i^—C14—C15^i^	162.3 (9)	H17C—C17—H17D	109.5
C16^i^—C14—C15	163.1 (8)	C15^i^—C18—C14^i^	66.7 (6)
C17^i^—C14—C15	121.3 (6)	C15^i^—C18—C17	121.8 (7)
C18^i^—C14—C15	53.6 (6)	C14^i^—C18—C17	55.2 (6)
C15^i^—C14—C15	109.9 (6)	C15^i^—C18—H18A	120.0
C16^i^—C14—C14^i^	111.7 (9)	C14^i^—C18—H18A	173.2
C17^i^—C14—C14^i^	171.1 (7)	C17—C18—H18A	118.2
			
O2—S1—N1—C7	53.15 (18)	C9—C10—C11—Cl2	−178.61 (17)
O1—S1—N1—C7	−179.10 (15)	C10—C11—C12—C13	−0.8 (4)
C1—S1—N1—C7	−62.70 (17)	Cl2—C11—C12—C13	178.16 (19)
O2—S1—C1—C6	−1.61 (18)	C11—C12—C13—C8	0.4 (4)
O1—S1—C1—C6	−132.58 (15)	C9—C8—C13—C12	0.5 (3)
N1—S1—C1—C6	114.87 (16)	C7—C8—C13—C12	−175.8 (2)
O2—S1—C1—C2	177.49 (16)	C16^i^—C14—C15—C16	42 (2)
O1—S1—C1—C2	46.52 (18)	C17^i^—C14—C15—C16	−178.0 (6)
N1—S1—C1—C2	−66.03 (18)	C18^i^—C14—C15—C16	−176.6 (7)
C6—C1—C2—C3	−1.0 (3)	C15^i^—C14—C15—C16	10.8 (6)
S1—C1—C2—C3	179.91 (16)	C14^i^—C14—C15—C16	10.8 (6)
C6—C1—C2—Cl1	178.61 (15)	C16^i^—C14—C15—C18^i^	−141 (2)
S1—C1—C2—Cl1	−0.5 (2)	C17^i^—C14—C15—C18^i^	−1.4 (7)
C1—C2—C3—C4	1.1 (3)	C15^i^—C14—C15—C18^i^	−172.6 (6)
Cl1—C2—C3—C4	−178.52 (19)	C14^i^—C14—C15—C18^i^	−172.6 (6)
C2—C3—C4—C5	0.0 (4)	C16^i^—C14—C15—C14^i^	31 (2)
C3—C4—C5—C6	−1.2 (4)	C17^i^—C14—C15—C14^i^	171.2 (7)
C4—C5—C6—C1	1.3 (4)	C18^i^—C14—C15—C14^i^	172.6 (6)
C2—C1—C6—C5	−0.2 (3)	C15^i^—C14—C15—C14^i^	0.0
S1—C1—C6—C5	178.95 (18)	C18^i^—C15—C16—C14^i^	−164 (5)
S1—N1—C7—O3	7.2 (3)	C14—C15—C16—C14^i^	−12.2 (7)
S1—N1—C7—C8	−172.21 (14)	C18^i^—C15—C16—C17	−164 (5)
O3—C7—C8—C9	−154.7 (2)	C14^i^—C15—C16—C17	−0.8 (5)
N1—C7—C8—C9	24.7 (3)	C14—C15—C16—C17	−12.9 (9)
O3—C7—C8—C13	21.4 (3)	C15—C16—C17—C14^i^	0.9 (5)
N1—C7—C8—C13	−159.19 (19)	C15—C16—C17—C18	9.8 (8)
C13—C8—C9—C10	−0.9 (3)	C14^i^—C16—C17—C18	8.9 (6)
C7—C8—C9—C10	175.12 (19)	C14^i^—C17—C18—C15^i^	1.4 (7)
C8—C9—C10—C11	0.5 (3)	C16—C17—C18—C15^i^	−7.0 (10)
C9—C10—C11—C12	0.4 (3)	C16—C17—C18—C14^i^	−8.4 (5)

Symmetry codes: (i) −*x*+1, −*y*, −*z*+2.

### Hydrogen-bond geometry (Å, °)

**Table:**

*D*—H···*A*	*D*—H	H···*A*	*D*···*A*	*D*—H···*A*
N1—H1N···O1^ii^	0.82 (2)	2.13 (2)	2.951 (2)	176 (2)

Symmetry codes: (ii) −*x*+1, −*y*+1, −*z*+1.
